# Historical Continuity or Different Sensory Worlds? What we Can Learn about the Sensory Characteristics of Early Modern Pharmaceuticals by Taking Them to a Trained Sensory Panel.

**DOI:** 10.1002/bewi.202000009

**Published:** 2020-09-04

**Authors:** Nils‐Otto Ahnfelt, Hjalmar Fors, Karin Wendin

**Affiliations:** ^1^ Department of History of Science and Ideas Division of Pharmacognosy Department of Medicinal Chemistry Uppsala University Uppsala Sweden; ^2^ Department of History of Science and Ideas Uppsala University Uppsala Sweden; ^3^ Unit for Medical History and Heritage Karolinska Institutet Stockholm Sweden; ^4^ Food and Meal Science Faculty of Science Kristianstad University Kristianstad Sweden; ^5^ Department of Food Science University of Copenhagen Frederiksberg C Denmark

**Keywords:** Early modern medicine, sensory analysis, history of pharmacy, reproduction, reworking, history of taste, history of olfaction

## Abstract

Early modern medicine was much more dependent on the senses than its contemporary counterpart. Although a comprehensive medical theory existed that assigned great value to taste and odor of medicaments, historical descriptions of taste and odor appears imprecise and inconsistent to modern eyes. How did historical actors move from subjective experience of taste and odor to culturally stable agreements that facilitated communication about the sensory properties of medicaments? This paper addresses this question, not by investigating texts, but by going straight to the sensory impression, which certain substances convey. The aim is not to overwrite or rectify historical descriptions but to investigate whether modern methodologies for sensory assessment can be enlisted to understand the past. We draw on history of science for framing and research questions, pharmaceutical science for knowledge of pharmaceuticals and preparations, and food and meal science for assaying procedures and protocols. We show that sensory evaluation can yield precise descriptions that would not have been alien to early modern medicine makers. However, there are problems with translating descriptions of taste between different historical contexts.  By comparing contemporary descriptions of sensations with eighteenth‐century ones, the article discusses how sensory descriptions are highly dependent on context, and subject to historical change.

## 1 Introduction

Historical medicine was much more dependent on the senses than its contemporary counterpart. Theoreticians postulated that miasmas, i.e., odorous airs, were an important source of disease. Medical practitioners and patients alike considered the sensory experience of a medicament as strongly indicative of its medical effects. Galenic humoral pathology, the main system used by Europeans for diagnosis and prescription from antiquity into the nineteenth century, paid due consideration to the sensory qualities of substances. Galen held that the bodily fluids corresponded to four qualities of taste: blood was sweet, phlegm was salt, yellow gall was bitter, and black gall sour and sharp. Physiologically, the medical virtues of a given substance also corresponded to its ability to produce states in the patient which corresponded to basic elemental (i.e., water, earth, fire, air) qualities: dampness or dryness, and heat and cold respectively.^1^ Ideally, the substance should restore balance to the humors, as illness was caused by imbalance. The sensory characteristics of a substance such as its ability to cause a sensation of heat or cold in the mouth were understood to correspond to its physiological effects. However, there were well‐known exceptions to these rules. Early modern theorists deemed opium hot (as in spicy) to the sense of taste, but as extremely cold when ingested, because of its powerful soporific effect which slowed down bodily processes.^2^ As is evident from herbals and works of natural history from antiquity, the middle ages and the early modern period, many spices, herbs and other types of foodstuff doubled as medicines.^3^ This connection made the link between the visual appearance, taste, flavor and odor of a medicine and its medical virtues self‐evident as spices and herbs were readily associated both to culinary and medical practices. Simultaneously, when we study descriptions of odor, taste and flavor written before the nineteenth century, it becomes clear that the vocabulary for describing these sensations was, in comparison to present day standards, rather poor. From Aristotle onwards, philosophers as well as medical theorists favored the sense of sight as their prime source of reliable knowledge about the world. As observed by Steven Shapin, early modern “repertoires for describing smells and tastes of food were neither extensive nor very discriminating.”^4^

This presents us with a conundrum. On the one hand a comprehensive medical theory clearly existed, which assigned a great value to taste and odor of medicaments. On the other, the language used to describe these aspects of medicaments seems imprecise and inconsistent to us now. How then did historical actors move from subjective experience of taste, flavor and odor to culturally stable agreements that made it possible to communicate about the properties of substances used as medicines?^5^ The present paper addresses this question, not by investigating texts and descriptions of substances, but by going straight to the substances, and by investigating the sensory impressions that they convey. The aim is not to overwrite or rectify historical descriptions. It is to investigate whether modern methodologies for taste, flavor and odor assessment can be enlisted for the purpose of understanding the past.^6^

It is the early modern period and the eighteenth century that concerns us here. We can safely assume that early modern Europeans possessed a discourse on the sensory experience of medicaments. Assessment, the process of making judgments about the identity, properties and quality of pharmaceuticals according to established, agreed upon procedures, was a main concern of traders, apothecaries, prescribing medical practitioners and patients everywhere. It was regarded as necessity to safeguard against fraudulent substitution and the sale of inferior goods. There were several methods used to assess pharmaceuticals. The most important ones were the investigations of taste, odor, visual appearance and weight of substances. In addition, collections could be created to permit comparisons between samples, and simple chemical tests were sometimes made for specific substances.^7^ This was an important business, and a key part of establishing consumer's trust in medicines. Venice, a central depot in the trade in spice and medicine, subjected all imported substances to a process called the *gerbelatura*. Through it, three things were ascertained: the identity, quality and purity of the substances.^8^ Aside from direct inspection of goods, larger trading companies provided some guarantee of quality and a country‐of‐origin labeling of sorts. An example of this is provided by the Russian monopoly which controlled the overland route of trade between China and Europe. It was a major player in the trade of Chinese medical rhubarb, and due to its efforts to safeguard quality, the “Russian” rhubarb was generally considered the best that could be had. Another example is the Dutch East India Company, which operated and controlled the whole chain of production of several important products, such as nutmeg and cinnamon, which were both used as spice and medicine throughout the early modern period. Nevertheless, as soon as the goods were unloaded and sold, the risk of fraud increased.^9^ Trust in the integrity of suppliers could only go so far in the global system of trade of early modernity. At the bottom line, pharmaceutical substances had to be made to speak for themselves. For this to happen, direct sensory experience needed to be translated into language, images and tacit, or gestural, knowledge.

## 2 Reasons for Setting up the Experiment

The questions we seek answers for in the present study are the following:

1. Can we establish a contemporary protocol for sensory evaluation which may be regarded as analogous to early modern practices of assessing pharmaceuticals?

2. Is it possible to rely solely on the taste, flavor and odor of processed pharmaceutical trading goods to create fine‐tuned and generalizable descriptions that may be used to ascertain the sensory properties of substances?

3. Can medically relevant knowledge of a substance be collectively construed, through linguistic description of sensory qualities?

These questions derive both from discussions when the study was designed and from previous work on reproducing early modern medicines by two of the authors of this study, Ahnfelt and Fors. It was this previous work that drew our attention to the striking and often highly characteristic taste and odor of both simple substances and composite medicaments that were used during the early modern period.^10^ To make this sensory experience more widely available we began to organize sessions where we invited participants to partake in the taste and odor of a selection of commonly used early modern pharmaceuticals. One of these sessions, presented as a plenary lecture at the conference *Materia medica on the move 2nd edition* in Amsterdam (2017), was filmed by director Katrien Vanagt and is available on YouTube.^11^ The movie illustrates well the surprise, appreciation and disgust that these substances may evoke when consumed. However, these experiences – even those that were so beautifully recorded by Vanagt and her team – remain personal and subjective; we therefore reflected on how to render the tacit explicit. How was individual sensory experience of pharmaceuticals translated into language and symbols that could be shared and agreed upon? To quote Shapin again, how would we get at the “collectively agreed‐upon procedures to notice, to describe, and to evaluate” which presumably were at play in early modern assessment and composition of medicaments.^12^

Philip M. Teigen identifies three contemporary methodologies to ascertain taste that may be said to produce judgments, or evidence, analogous to Galenic pharmacology's systematic use of taste. They are 1) the psycho‐physiology of taste, i.e., the assignment of taste to specific chemical compounds that come in contact with the sensory organs, 2) sensory evaluation and 3) technologically‐assisted analysis.^13^

Of these three methodologies it is undoubtedly the second path, sensory evaluation, that bears the closest similarities to the practices of assessment carried out by early modern apothecaries. Knowing that early modern pharmaceutical products were both medical objects and consumer goods, we sought the help of a specialist in transferring individual experience into language and symbols intended to facilitate evaluation of consumer goods, i.e., food and meal scientist Wendin. Teaming up with Wendin to transfer tasting sessions from the congenial environment of the scientific gathering to that of the more formal scientific study also made it necessary to scrutinize the issue of safety protocols (more on this below).

**Figure 1:**
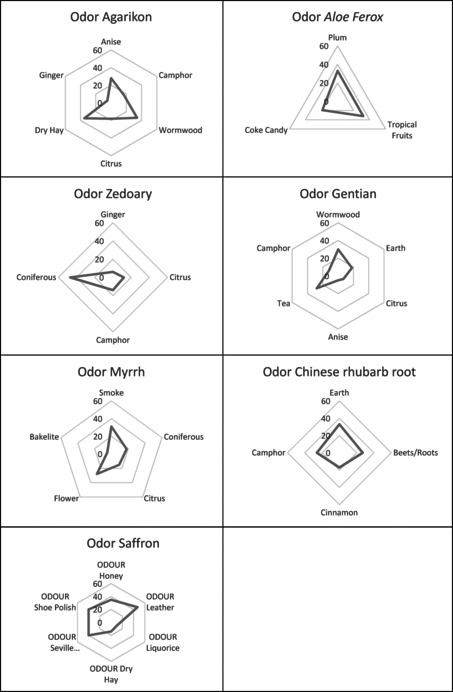
Odor profiles of the pharmaceuticals.

**Figure 2:**
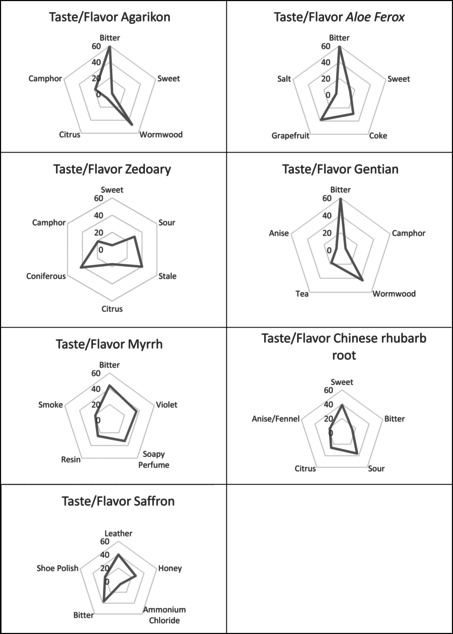
Taste and flavor profiles of the pharmaceuticals.

The experiment we came up with makes use of sensory analysis by means of a panel of trained assessors to investigate odor, flavor and taste of seven botanicals commonly used as medicines during the early modern period and the eighteenth century. The method used by assessors is a slightly modified version of the Flavor Profile Method® invented by Arthur D. Little. The method entails that a small group of assessors (in our case eight) are instructed to identify and describe the sensory attributes (taste, flavor and odor) of samples put before them. Assessors propose attributes and then discuss until they reach a consensus on which suggested attributes should characterize each sample, whereupon the assessors assign each attribute a value on an intensity scale running from zero to 100.^14^ When the resultant data have been processed, the results can be presented as a list of descriptions or as a graph (see Table 2 and 3, and Figure 1 and 2).

The chosen botanicals were agarikon (*Laricifomes officinalis*), cap aloe (*Aloe ferox*) zedoary (*Curcuma zedoaria*), gentian (*Gentiana lutea*), myrrh (*Commiphora molmol*), Chinese rhubarb (*Rheum palmatum*) and saffron (*Crocus sativus*). They were chosen because we knew from previous work that they are non‐toxic, have highly distinctive sensory profiles and are (with the exception of saffron) largely unknown entities in the present day and age. Furthermore, they were popular medical components all over Europe throughout the early modern period. Among the recipes in a representative late seventeenth‐century pharmacopeia, such as the *Pharmacopeja Holmiensis* (1686), aloe (*Aloe ferox*) was used in 18.4 % of the recipes, and myrrh in 7,7 % of the recipes. They were also popular substances in combination: 20 formulations out of the total 494 formulations found in *Pharmacopeja Holmiensis* contained three or more of these seven simple plant substances. Representative composite medicaments are, e.g., *Pilulae de Agarico* which contained myrrh, agarikon, saffron and aloe, and *Massa Polychresta* which contained zedoary, aloe, myrrh, saffron and another 13 ingredients.^15^

The substances represent a wide spread of geographical sources, and plant parts. All of them are made from processed and dried parts of plants. Gentian, zedoary and Chinese rhubarb are made from roots or rhizomes. While gentian is native to Europe, zedoary is native to Southeast Asia, and Chinese rhubarb to southern China and Tibet. Aloe and myrrh are plant resins, originating in southern and northern Africa, respectively. Agarikon is made from the fruiting body of a parasitic fungus which is native to Europe.^16^ Saffron consists, as most people know, of the stigmas and styles of a flower native to the Middle East and particularly Persia (Iran). Some, if not all substances, have been in use by humans for thousands of years. For example, the use of saffron dates back to ancient Persia and rhubarb root has been in use for several thousands of years in China.^17^ Taken together, the substances represent the wide variety of places and plants from which early modern pharmaceuticals were sourced. The majority, i.e., zedoary, Chinese rhubarb, aloe, myrrh and saffron, were very expensive, and hence prime candidates for counterfeit during the early modern period.

## 3 Practical, Ethical, and Historical Considerations

We chose not to investigate visual appearance of substances, nor the sensation of texture or *mouthfeel*. The reason is that from a sensory point of view, it is not color and texture but odor and taste that most clearly distinguishes botanicals used as pharmaceuticals from each other. With some notable exceptions (such as indeed bright yellow‐red saffron) most dried and processed botanicals used for pharmaceutical purposes come in various shades of brown. Choosing not to study visual properties was also necessary to keep the study within reasonable bounds. Although interesting, a study of, e.g., the appreciation of the colors of botanicals would be very different from the present one and would need to draw on a completely different set of sources. Structure, texture and mouthfeel is also not studied for similar reasons. It is however known that these were used to assess pharmaceutical substances. One of our test substances, Chinese rhubarb root, was well‐known for its gritty mouthfeel when chewed, as if it contained a fine‐grained sand.^18^ Hence, it would have been relevant to incorporate mouthfeel in the present study. But there is scant historical literature on the topic and taking this aspect of sensation into account would also have required us to use a different experimental setup.

In order to reduce exposure to substances to a minimum, our samples consist of alcohol extracts (tinctures) made from each of the substances. There is a certain historical validity to this approach, as the evaluation of the potency of tinctures would have been a specific challenge facing apothecaries. But this also means that we chose not to reproduce or reenact methods used in the past for assaying unprocessed seed, gum or bark, such as chewing pieces of substances or inhaling them in powdered form. Safety is tantamount, and putting physiologically active substances, i.e., spices and medicines, in the mouths of people (such as students) is not something that historians should just start doing. There are many potential dangers, not just concerned with potential toxicity issues. Many of the substances historically used in pharmacy are, in the present day and age, rare or completely disused as medicines or foodstuffs, and test persons may not be expected to have come across them before trials. This means that it is advisable to reduce exposure to a minimum to reduce the risk of harmful effects, such as allergic reactions. It is also necessary to have access to competence in pharmaceutical science/toxicology, and to proceed from such safety protocols as have been developed in food science for the administration of foodstuffs to human subjects. Experiments of this type are in fact experiments on humans. In practice it means that human subjects, e.g., assessors in a sensory panel, must be informed about the substances they are analyzing and give their informed consent of participation. Participation should be voluntary and the assessors should have the right to leave the study without explanation.

We understand that our choice to use the protocols provided by modern food science has taken us quite far from the practices of historical actors as described in historical sources. The readers may decide themselves how valuable this approach is for enhancing our understanding of the history of sensory practices. Yet we think that an experimental multidisciplinary approach to sensory history such as this one helps to explore the possibilities and limitations of performative methods in the history of science and knowledge. This study draws on history of science for framing and research questions, on pharmaceutical science for knowledge of pharmaceuticals and preparations, and on food and meal science for assaying procedures, safety measures and protocols. Historians rarely possess knowledge in these latter areas, and the respective skills are not easily acquired; they need to collaborate with specialists from other fields. They may also find the results produced in other disciplines useful to their purposes. The protocols used in food science, for example, aim to transfer sensory experience into standardized graphs and descriptions, which can be utilized in historical studies for further comparisons and investigations. As the present study shows, some of the findings in modern food science can address historical questions that otherwise would have remained unanswered.

We must nevertheless acknowledge the limits of studies such as this one. It cannot be taken for granted that substances that are available today can be used to represent early modern substances, or that our experiences of them are similar to those of historical actors. Traded medicinal substances, although they appear to be products of nature, are cultural constructs to a much greater degree than is generally acknowledged. Every single substance may have small or big variations in almost every parameter. Plants that were harvested wild or semi‐wild may now be available only as cultivated and might now be grown in other places and climates as compared to when they were originally harvested; ecosystems may have changed. Important knowledge such as the correct season to harvest as well as which plant parts to use may have changed or is no longer available. The same applies to the modes of cultivation and harvesting. The means of transport and storage have dramatically changed since early modern times. Notions about purity of substance have been transformed. There may be ways to avoid this conundrum, but their development goes beyond the scope of the present paper and the larger research project of which it is a part. We study and attempt to reproduce the experiences, knowledge and skills of early modern apothecaries, while acknowledging that this cannot be done with absolute historicity. Like the historical actors, we work both with texts and with processed, traded goods, and do not have the resources to conduct substantial investigations into supply chains and methods of cultivation.^19^

Such caveats aside, we argue that this work nevertheless provides new access to the early modern world and how we perceive it. Experiencing pharmaceutical substances does not revive historical pharmacy as a sensory experience, it also enables us to comprehend it as a knowledge practice. By developing protocols for the multi‐sensory experience of historical objects (such as medicaments, spices and drinks) we create a space for these objects in contemporary life, and it thus becomes possible to talk about them as multi‐sensory objects, not just as textually produced artifacts. Our findings may be of interest for historians of early modern pharmacy and medicine and historical bio‐prospectors investigating traditionally used medicaments, as well as for historians and pharmaceutical researchers more generally.

## 4 Materials and Methods

The seven pharmaceuticals selected were obtained from a commercial supplier, Galke GmbH, Germany. They are described in Table 1.

**Table 1:** The seven studied pharmaceuticals with Latin names, major chemical constituents and contemporary pharmaceutical usage; citations from respective page in Evans 2009 and Grienke et al. 2014.

Pharmaceutical (*Latin name of plant of origin*)	Odor sample (w/v)	Oral sample (w/v)	Chemical constituents	Properties and usage
Agarikon (*Laricifomes officinalis*)	4 %	0,25 %	Triterpenoids (Grienke et al. 2014)	Bitter taste, antiviral
Cap aloe (*Aloe ferox*)	4 %	2 %	Aloeresin A, B and C, p. 248	Stimulant laxative, p. 491.
Zedoary (*Curcuma zedoaria*)	4 %	0,125 %	sesquiterpenoids, p. 442	Carminative, spicy and digestive
Gentian (*Gentiana lutea*)	4 %	0,125 %	Gentiopicroside, amarogentin, p. 334	Extremely bitter, p. 335
Myrrh (*Commiphora molmol*)	4 %	2 %	Terpenes, esters, cuminic aldehyde and eugenol, p. 298–299	Incense and perfume, astringent
Chinese rhubarb root (*Rheum palmatum*)	4 %	0,5 %	Anthraquinones and its glycosides, p. 244–246	Bitter stomachic, both purgative and astringent
Saffron (*Crocus sativus*)	4 %	0,25 %	Picrocrocin, crocin and safranal, p. 474	Chinese medicine; antioxidant

Solutions of each ingredient were made through extraction of each ingredient using 40 % v/v ethanol (Absolut Vodka, Åhus, Sweden) to a content of 4 % w/v, respectively, in brown flasks with a glass stopper. Extraction commenced for one week at a temperature of approximately +10 °C and each flask was shaken every day for a few seconds. After filtration through a coffee filter paper the test samples were prepared according to the following:

Odor samples for sensory analysis were prepared at a 4 % (w/v) content. The samples were prepared by soaking a cotton pad with about 5 ml test solution into an aluminum box (15 ml volume) equipped with an aluminum screw lid (Table 1). Oral samples (taste and flavor) of 5 ml of each ingredient were added into 10 ml brown glass flasks with an aluminum stopper.

All test samples were prepared at the division of Pharmacognosy, Department of Medicinal Chemistry, Uppsala University, Sweden and taken by train to Kristianstad, Sweden, where sensory analysis was performed at the Department of Food and Meal Science, Faculty of Science, Kristianstad University. Although temperature changes were not deemed to influence taste, flavor or odor of test samples in alcohol solution, samples were stored in a refrigerator (+4 °C) upon arrival and accommodated to room temperature for one hour before sensory analysis as part of a standardized procedure. Reference samples were prepared in beakers with lids in order to help the sensory panel to identify tastes and odors in the samples. All references were bought at the supermarket *ICA* in Kristianstad, Sweden. The reference samples were: Fresh lemon, fresh lemon peel, fresh ginger (minced), eucalyptus pastilles for managing cold (Vicks® Blue, extra strong, Procter and Gamble, USA), dry juniper berries (Kockens kryddor, Lyckeby Culinar, Sweden), fresh mint leaves (*Mentha spicata*), fresh grapefruit, fresh lime, clove and allspice (Kockens kryddor, Lyckeby Culinar, Sweden), Swedish Christmas non‐alcoholic carbonated drink (“Julmust,” Apotekarnes, Sweden) and honey (Svensk Biodlarförening, Sweden; diluted in some warm water for ease of consumption).

The samples were analyzed by a slightly modified version of the Flavor Profile Method® invented by Arthur D. Little.^20^ The modification consisted of the use of a scale running from zero to 100 instead of the shorter scale used in the original method. The sensory panel consisted of eight assessors who were selected and trained according to ISO standard 8586–2:2008, and who have highly sensitive senses of taste and olfaction.^21^ The assessment lasted for approximately four to five hours divided into two sessions during two consecutive days, meaning that each sample was assigned between an hour and an hour and a half. Before assessing samples, the panel rehearsed how to perform testing and how to judge intensity on a numerical intensity scale, running from zero to 100. Reference samples and a few selected odor test samples extracts were assessed until consensus reached before the main assessing of the ingredients took place.

Each extract was assessed “one by one,” first the smell from the odor sample and then taste and flavor from the oral sample. The assessors were instructed to identify and describe the sensory attributes, taste, flavor and odor, in each test sample until consensus was reached upon a definition for each attribute. Then each attribute was assessed using the intensity scale. The assessors were instructed to use the full length of the scale in order to be able to distinguish between samples which only slightly differed from each other. Each assessor needed to agree upon the placement of each attribute along the intensity axis. Between each sample the sensory panel had a break for ten minutes to refresh their senses. The assessors were instructed to use water and neutral wafers to clean their palate and neutralize their senses. As part of the safety protocol, the assessors were informed not to swallow the samples and to spit out right after assessment. Furthermore, the panelists were informed about the samples and their pharmacological potential as pharmaceutical ingredients using information from a standard textbook in pharmacognosy before agreeing to join the assessments.^22^ Each assessor signed for participation after being informed about the products and the terms for participation, which meant voluntary participation, freedom to leave the test without giving a reason, the right to decline to answer specific questions and an assurance that their participation would not affect their future treatment in the health care system in any way.

## 5 Results and Discussion

The results show that all seven pharmaceuticals had rich and specific odor and oral profiles. The perceived odors are described in Table 2 and the tastes and flavors in Table 3.

**Table 2:** Odor attributes, definitions and in which sample they were perceived.

Attribute	Definition	Perceived in
Ginger	Specific trigeminal	Zedoary*, agarikon
Honey	Honey wax	Saffron
Wormwood		Gentian, agarikon
Camphor	Minty and menthol‐like	Chinese rhubarb root, gentian, zedoary, agarikon
Leather	New shoes	Saffron
Liquorice	Sweet liquorice	Saffron
Dry Hay	Dry hay from hayloft	Saffron, agarikon
Seville Orange	Old English Marmelade	Saffron
Shoe Polish	Petroleum, pesticide	Saffron
Earth	Earthy cellar	Chinese rhubarb root, gentian
Beets/Roots	Blend of edible roots/beets	Rhubarb root
Cinnamon		Rhubarb root
Smoke	Burnt wood, old whisky	Myrrh
Coniferous	Pine and fir	Myrrh, zedoary
Citrus	Blend of citrus fruits	Myrrh, gentian, zedoary, agarikon
Flower	Lavendel, rose	Myrrh
Bakelite	Old plastics	Myrrh
Anise	Liquorice, fennel, dentist	Gentian, agarikon
Tea	Stale black tea	Gentian
Plum	Blend of Nordic fruits plum, rhubarb, apple	*Aloe ferox*
Tropical Fruits	Blend of Tropical fruits pine apple, banana, carambole, citrus	*Aloe ferox*
Coke Candy	Soft candy flavored with coke, fruits	*Aloe ferox*

* In zedoary the perception of ginger is a short and initial

**Table 3:** Oral attributes, definitions and in which sample they were perceived.

Attribute	Definition	Perceived in
**Taste**		
Sweet	Sweet taste	Chinese rhubarb root, zedoary, *Aloe ferox*, agarikon
Bitter	Bitter taste	Saffron, Chinese rhubarb root, zedoary, myrrh, gentian, *Aloe ferox*, agarikon
Sour	Sour taste	Chinese rhubarb root, zedoary
Salt	Salty taste	Aloe ferox*
**Flavor**		
Coke candy	Soft candy flavoured with coke, fruits	*Aloe ferox*
Violet	Flower mainly violet	Myrrh
Camphor	Minty and menthol‐like	Gentian, zedoary, agarikon
Leather	New shoes	Saffron
Honey	Honey wax	Saffron
Ammounium chloride	Liquorice like	Saffron
Shoe polish	Petroleum, Pesticide	Saffron
Grapefruit peel	Citrus with distinct flavour of grapefruit peel	Chinese rhubarb root, *Aloe ferox*
Anise	Liquorice, Fennel, Dentist	Chinese rhubarb root, gentian
Soapy perfume	Perfume common in soap	Myrrh
Resin	Specific from pine and fir	Myrrh
Smoke	Burnt wood, old whisky	Myrrh
Wormwood		Gentian, agarikon
Tea	Stale black tea	Gentian
Stale	Old tree	Zedoary
Coniferous	Pine and Fir	Zedoary
Citrus	Blend of citrus fruits	Agarikon

* Perceived as an after‐taste

It is noteworthy that unexpected odors and flavors occurred in some of the ingredients. For example, ginger was perceived as an odor attribute of agarikon and zedoary. In the literature, only the flavor of zedoary has been described to be reminiscent of ginger, along with a bitter aftertaste.^23^ It is also well known that the Asian kitchen uses zedoary and sometimes replaces ginger by zedoary.^24^ Both zedoary and ginger, along with other spices, belong to the genus *Curcuma* of the family *Zingiberaceae*. In this study ginger is noted as a distinct odor (Figure 1).^25^ By studying the perception of the ingredients further, it can be seen that camphor is perceived in Chinese rhubarb root, gentian, zedoary and agarikon, both as flavor and odor (Tables 2 and 3). Camphor is found in the Asian “camphor tree,” but also in different herbs and spices. Camphor is a terpenoid and is perceived especially as an odor (Figures 1 and 2).^26^ Citrus is perceived as a non‐distinct odor and/or flavor in myrrh, gentian, zedoary and agarikon (Tables 2 and 3 and Figures 1 and 2). Citral and geranial, causing the citrus flavor, belong to the terpenoids and are found in several plants and herbs. Citral has since long time been used as a masking agent for unpleasant flavors and odors.^27^

Bitterness and sweetness are the most profound tastes in the assessed ingredients (Figure 2). As seen in Table 1 most ingredients are described as having a bitter or very bitter flavor (see also Table 3). This corresponds to descriptions in historical sources. Bitterness was an important feature when assessing potential herbals as medicine already in ancient pharmacology.^28^ This was acknowledged also in the early modern period. According to Carl Linnaeus, bitter substances were important in medicines as well as including sweet substances like honey.^29^

When comparing the pharmaceuticals’ sensory qualities with each other, it becomes obvious that profiles differ widely. Gentian, for example, comes across as extremely bitter in taste, and is indeed a well‐known bittering agent nowadays, which is used in alcoholic digestives. All ingredients were described to have intense taste, flavor and odor. Figures 1 and 2 show the sensory profiles of each ingredient. These ingredients differ significantly from each other, although as mentioned above they have some attributes in common.

With regards to taste and flavor, on the basic, *attribute* level of description (e.g., salt, sour, bitter etc.: Table 3), the intense tastes show a strong consistency with descriptions in historical sources. However, when we go deeper into the descriptive terminology used to characterize the sensory experiences and compare with descriptions in historical sources, differences become apparent. This can be illustrated by comparing the descriptions above with those of the world‐renowned botanist and physician Carl Linnaeus (1707–1778). Linnaeus relied exclusively on taste, odor and flavor to assess pharmacological potential of medicinal herbals. This is evident from at least eighteen of his 186 dissertations during 1747–1776.^30^ In his book *Clavis Medicinae Duplex* (“The two keys of Medicine”), sensory qualities were important in the assessment of herbal drugs possible pharmacological action.^31^ Odors are characterized as five opposing pairs of sweet‐smelling and evil‐smelling, with respective examples given within parenthesis: ambrosiac (musk)—rank‐smelling (he‐goat); fragrant (lily)—reeking (hemp); sweet‐scented (cat thyme)—stinking (opium); aromatic (cinnamon)—nauseating (colocynth); orgastic (garlic)—pungent (henbane). Taste was subdivided into four different classes: watery, dry, sour and bitter. In the somewhat later dissertation *Medicamenta purgantia* (Purging medicaments) from 1775, the stinking odors are subdivided: *hircosa* (goat‐smelling), *nidorosa* (smoky), *tetra* (disgusting), *nauseosa* (nauseous), and *virosa* (sharp, pungent). The treatise dealt with purging medicines that Linnaeus was very appreciative of, e.g., “in order to free the intestinal canals of undigested material, to rid the body of detrimental and superfluous elements”.^32^ Among the medicaments found as examples we observe Agaricus (*Laricifomes officinalis*), Aloe (*Aloe ferox)* and Rhabarbarum *(Rheum palmatum)* three of the substances investigated in the present study.

As is apparent from Linnaeus's work, detailed sensory analysis of pharmaceuticals was not unknown during the early modern period. There is also a certain historical continuity in basic descriptive terminology; both Linnaeus and the taste panel use basic descriptive terminology such as bitter, and sweet, but also more precise words (i.e., smoky) are shared. We may conclude that if, e.g., an eighteenth‐century source claims that a drug is bitter or sweet in taste, we would agree. This should not come as a surprise. It would be strange to completely deny the cultural and linguistic continuity that connects us with our near ancestors, while biologically, of course, human bodies have barely changed at all.

When it comes to more detailed levels of description, however, the situation is different. It becomes apparent that late moderns and early moderns inhabit different sensory worlds. It is difficult for most late moderns to appreciate how the smell of goat is different from a disgusting smell, or from a sharp/pungent smell. Similarly, Linnaeus could only have guessed at what is meant by characterizing saffron as smelling of *shoe polish* and *ammonium chloride* and myrrh as smelling of *bakelite* and tasting like *soapy perfume*.

The human senses, and consequently human ability to distinguish between different tastes and odors, do not change over such short timespans as a couple of hundred years. Nevertheless, what this study shows is that the reference points used to distinguish sensory information are highly dependent on culture. Many of the descriptions used by the test panel would be incomprehensible to early moderns in whose culture the objects referred to did not exist (bakelite, plastics, pesticide), would have smelled very different (shoe‐polish) or would have been extremely rare (petroleum). Consequently, we may surmise that the same is true also when we read early modern descriptions of smell and taste. It is very likely that there are associations and references that are simply inaccessible to us. One such associative world which seems to have closed, is the medieval and early modern associations of sample plants with places of worship, pharmacies, the Orient, as well as opulence, rarity and wealth.^33^ No such terminology whatsoever come across in the panel's descriptions. The most likely explanation for this is that substances no longer carry such associations (as in the case of, e.g., saffron) or that substances that retain religious associations (e.g., myrrh) have fallen out of general use (at least in Protestant Sweden, where the assaying was conducted). It may indeed be possible that the panel would have associated myrrh with religion and church service if they had realized that it was myrrh that they sampled. The test panel was however not given access to any historical or other background information about the samples.

Another world of association and description that, surprisingly enough, was barely accessed is that of medicine. Indeed, given that early moderns considered all these tested substances to be medicaments, the lack of attributes and definitions referring to medicine is striking. There is just one example: the attribute anise defined as smelling of dentist was perceived both in gentian and agarikon (Table 3). This point to a strong historical discontinuity with respect to cultural perceptions of the use of spices and exotic botanicals as medicines.

However, early modern sensation and precision in description was no less acute than that of present times. The assessors’ ability to assess differences between sample substances, and to articulate them in precise terms, may be generalized. It should be noted that the assessors did not show that their evaluations were reliable between multiple encounters with the same substance. The assessors are, however, no experts in recognizing a certain drug or specific product. Their expertise lies in identification and quantification of descriptors/attributes of the samples. And this is, arguably, precisely the skill that was necessary for early modern apothecaries sampling the quality of trade goods, stocked products or composing composite medicines. In order for a composition to be recognizable as, e.g., *theriac andromachalis*, *mel aegyptiacum*, *syrupus zingiberis* or whatnot, it was necessary that it should have consistent attributes with regards to taste, flavor and odor. Even though medicinal recipes typically are highly precise in their instructions on which quantities of substances to use, apothecaries still had to draw on their senses to check the outcome of mixtures, as the strength of the ingredients could vary. In order to achieve consistent results in medical composition, sensory profiles would have been of utmost importance. The sensory experience of both ingredients and blended medication was valued by medical practitioners as well as patients.^34^ Hence our study supports the position that sensory evaluation of odor and taste/flavor was a necessary and readily available tool for assessment of medicinal herbals and composition of medicaments as performed by the early modern apothecary.

## 6 Concluding Discussion

In this paper, we ask three questions: Can we establish a contemporary protocol for sensory evaluation which may be regarded as analogous to early modern practices of assessing pharmaceuticals? Is it possible to rely solely on the taste, flavor and odor of processed pharmaceutical trading goods to create fine‐tuned and generalizable descriptions that may be used to ascertain the sensory properties of substances? Can medically relevant knowledge of a substance be collectively construed, through linguistic description of sensory qualities?

In our view, all three questions can be answered in the affirmative. We know that odor and taste/flavor played important roles in ancient, medieval and early modern medical theory. This study shows the richness of the sensory profiles of seven commonly used early modern pharmaceuticals. Using a strict protocol and under controlled conditions, the sensory profiles of each ingredient were characterized, defined and distinguished from other ingredients by a temporary knowledge collective: our sensory panel. The study shows that odor and taste/flavor can be used, even by someone who is unfamiliar with the specific substances, to assess and distinguish between attributes of early modern herbal drugs in a highly precise way, yielding both detailed and precise information about pharmaceuticals. Furthermore, we show that this process can be enacted as a collective process, where all participants reach an agreement on the properties of the sampled substances. This suggests that individuals working within the artisanal setting of the early modern pharmacy, who had both longer exposure to substances, and the opportunity to talk and interact with senior apothecaries about them, should perform even better. We may thus conclude that it is highly likely that early moderns were able to identify crucial taste, flavor and odor attributes that were expected to be present in pharmaceutical plants. Conclusively, we may also consider processed goods as stable and comparably secure objects of knowledge to the early modern apothecary, something that should not be taken for granted. Sensory assessment of odor and taste/flavor, complemented with visual inspection were therefore, most likely, highly effective and precise tools for apothecaries making assessment of drugs. These “sensory tools” used along with verbal descriptions could be used in assessing whether herbal drugs were of the right quality, whether they were counterfeits or perhaps stored or transported too long or under poor storage conditions. They were also most likely important in making decisions about composition of medicines. Taste, flavor and odor, therefore, were likely to have a real and valuable function as tools in the arsenal of the apothecary.

This study indicates that medicine making was a much more sensory experience than existing research on the history of pharmacy assumes. It underlines the importance of taking into account odor, taste and flavor when studying pharmaceuticals used in ancient, medieval and early modern medicine. However, caution must be exercised: descriptions of taste, flavor and odor are highly dependent on context, and they are subject to historical change. As we have noted, the sensory panel used a descriptive terminology, which to a significant extent would have been incomprehensible to early modern people. Simultaneously, there was no or little tendency to situate the samples by means of exoticizing, religious or medicinal descriptive terminology. Given that all these tested substances were regarded as important and powerful herbal drugs by early modern apothecaries and physicians, this clearly points to a deep historical disruption in our cultural expectations about the odor and taste of medicines.

By developing a protocol for bringing experiences of historical materials (such as medicaments, spices, and drinks) into modern life, the study also emphasizes the benefits of a multidisciplinary approach. We have argued strongly for the necessity of appropriate safety measures and protocols when conducting studies of this kind. Most historians are not familiar with these bodies of knowledge, and, hence, should collaborate with specialists from other fields. This also implies that, at least to some extent, historians need to let people with other types of expertise lead the way, be they scientists, artist, curators or artisans. Historians (and archaeologists) should not attempt to claim ownership of the fields of historical reworking and reproducing. For the field to develop, it may often be necessary to work multidisciplinary, and this means accepting and going along with theories, methods and agendas current in other professional groups. It is after all possible to return to one's own turf whenever one wants to.

## Footnotes

1 Palmer 1993; Corbin 1994; Mandelkern 2015.

2 Teigen 1987; Klerk 2014.

3 Anderson 1977.

4 Shapin 2011. Quotation on 13–14.

5 Tastes are perceived by taste receptors in the mouth (on the tongue and in the oral cavity) and odors are perceived by odor receptors placed in the nasal cavity. The perception of flavors is mediated by retronasal aromas taking their path from the oral cavity where odor receptors are activated.

6 Ahnfelt and Fors 2016; Fors et al. 2016.

7 Pugliano 2017; Maranta and Camerarius 1576.

8 Schmitz 1998.

9 Foust 1992; Czarra 2009.

10 Ahnfelt and Fors 2016.

11 https://www.youtube.com/watch?v=lLReaTxwhEQ.

12 Teigen 1987; Shapin 2016. Quotation on 442.

13 Teigen 1987.

14 Lawless and Heymann 2010.

15 [Ziervogel] 1689.

16 Pegler 2000.

17 Kafi et al. 2006; Foust 1992.

18 Scheele 1784.

19 Ahnfelt and Fors 2016.

20 Lawless and Heymann 2010.

21 ISO standard 8586–2:2008: Sensory analysis—General guidance for the selection, training and monitoring of assessors—Part 2: Expert sensory assessors.

22 Evans 2009.

23 Everett 1982, on 3591.

24 Sutarno et al. 1999.

25 Mann et al. 1994, on 309–311.

26 Lincoln and Lawrence 1984; Fenaroli et al. 1975.

27 Lawless and Heymann 2010; Lim 2015.

28 Hart 2005.

29 Linnaeus 2012.

30 Nynäs and Bergquist 2016.

31 Linnaeus 2012.

32 Linnaeus and Rotheram 1775.

33 Freedman 2008.

34 Ahnfelt and Fors 2016; Linnaeus 2012.
